# Untargeted Metabolomics of Human Airway Epithelium Reveals Neuroactive Signatures Linked to Pulmonary Neuroendocrine Cell Enrichment and Allergen Exposure

**DOI:** 10.3390/metabo16020137

**Published:** 2026-02-17

**Authors:** Ritu Mann-Nüttel, Ayshna Diya, Paul Forsythe

**Affiliations:** Division of Pulmonary Medicine, Department of Medicine, Faculty of Medicine & Dentistry, and Alberta Respiratory Centre, University of Alberta, Edmonton, AB T6G 2S2, Canada; mannnutt@ualberta.ca (R.M.-N.); ayshna@ualberta.ca (A.D.)

**Keywords:** house dust mite, metabolomics, neurotransmitters, pulmonary neuroendocrine cells

## Abstract

**Background:** Pulmonary neuroendocrine cells (PNECs) are rare airway sensory cells implicated in amplifying allergic inflammation, yet due to their scarcity, the contribution of PNECs to the metabolic programs and responses of the airway epithelium remains poorly defined. Using a newly developed PNEC-enriched human airway epithelial model (ePNEC), we investigated the influence of PNECs on neuroendocrine and immune-modulatory metabolite production in response to the common aeroallergen of the house dust mite (HDM). **Methods**: Human bronchial epithelial cells (HBECs) and ePNEC cultures were differentiated at the air–liquid interface. Global untargeted metabolomics was performed to quantify metabolite abundance at baseline and following stimulation with HDMs. Differential expression, overlap significance, metabolite class enrichment, and pathway analyses were used to define PNEC-specific metabolic programs. **Results**: Principal component analysis (PCA) demonstrated strong baseline separation between ePNECs and HBECs, with HDMs inducing additional within-cell-type shifts. ePNECs displayed broader and more pronounced metabolite changes than HBECs. Baseline differences were largely preserved following allergen exposure, with significant overlap in both up- and down-regulated metabolites. ePNECs exhibited enriched neurotransmitter-linked metabolites—including serotonin, L-noradrenaline, dopamine, and histamine—at baseline and after HDM exposure. Amino acid–centered metabolism dominated the dataset, with enhanced histidine and tryptophan pathway activity in ePNECs. Pathway analysis revealed significant enrichment of phenylalanine, tyrosine, tryptophan, glutathione, and arginine–proline metabolism in ePNECs, whereas HBECs showed no significant pathway-level enrichment after HDM exposure. **Conclusions**: Human ePNECs engage a distinct, neuroactive metabolic program that is amplified upon HDM exposure. These findings provide a metabolic framework for how PNECs shape epithelial and neuroimmune responses to inhaled allergens.

## 1. Introduction

Pulmonary neuroendocrine cells (PNECs) are rare sensory cells that appear either as solitary cells dispersed along the airway epithelium or as small clusters located at airway branch points [1]. They are characterized by dense-core secretory vesicles containing a range of neuropeptides and neurotransmitters, including calcitonin gene-related peptide (CGRP), γ-aminobutyric acid (GABA), and serotonin [2,3,4,5]. Interest in PNECs has grown recently due to their proposed role in amplifying allergic airway inflammation [6]. In murine models of asthma, PNEC-derived CGRP has been shown to activate group 2 innate lymphoid cells (ILC2s), promoting downstream type 2 immune responses [6]. In humans, asthma is similarly associated with increased PNEC numbers [6]. Despite these insights, our understanding of human PNEC biology and their responses to asthma-relevant stimuli, such as the house dust mite (HDM) allergen, remains limited. This gap persists largely because PNECs constitute only a tiny fraction (approximately 0.04%) of the airway epithelium [7] and cannot be readily isolated using existing methods. To address this challenge, we recently established a novel model of a PNEC-enriched human airway epithelium (ePNEC) generated from primary bronchial epithelial cells (HBECs). This in vitro model contains ~ 20% PNECs and other cell types such as goblet cells, club cells, basal cells, ciliated epithelial cells, and myofibroblasts representative of the lung environment [8]. We found that the ePNECs respond to HDM challenge by the production of CGRP, a neuropeptide, by signaling through the protease-activated receptor 1 (PAR1) [8]. As traditional quantitative real-time PCR cannot directly measure levels of neurotransmitters, which are not transcribed from genes but synthesized directly from amino acids, we used untargeted metabolomics to profile global small-molecule changes in ePNEC versus HBEC cultures at steady state and after short-term HDM exposure. Untargeted metabolomics provides an unbiased survey of diverse biochemical classes (amino acids, biogenic amines, organic acids) and therefore captures neurotransmitters and their precursors that transcript-based methods miss. This makes it well suited to reveal neuroactive and immunomodulatory pathways such as purine and tryptophan metabolism that are known to modulate epithelial–immune communication in the lung [9]. Understanding these pathways is especially relevant in the context of allergic inflammation, where PNEC and epithelial cells serve as the first responders to inhaled allergens such as HDMs. Furthermore, we collected basolateral conditioned media rather than cellular extracts because basolateral secretions represent the compartment most relevant to epithelial–neural and epithelial–immune signaling. Basolateral release captures metabolites and signaling molecules that would interact with subepithelial nerve endings and immune cells, enabling detection of secreted or transported metabolites that mediate paracrine neuroimmune communication. Basolateral media from these cultures have previously been studied in a time course experiment, with transcriptional changes in response to HDM detectable by 2 h, whereas robust protein and neuropeptide release was most consistent at 6 h. We observed HDM-induced substance P and CGRP release from ePNECs and Interleukin-8 and Granulocyte–Macrophage Colony-Stimulating Factor (GM-CSF) release from both ePNECs and HBECs at 6 h [8]. Accordingly, we sought to assess the metabolomic profile at this time point. Using untargeted global metabolomics, we defined changes in metabolite levels and pathways in ePNEC compared to HBEC cultures at the air–liquid interface (ALI), both at steady state and following short-term HDM stimulation. This work provides a metabolic framework for understanding how ePNECs contribute to airway epithelial function and neuroimmune communication.

## 2. Materials and Methods

### 2.1. Cell Culture and Stimulation

ePNECs and HBECs cultured at air–liquid interface (ALI) were cultured as described in Mann-Nüttel et al. [8]. Both the HBEC and ePNEC cultures were derived from a single healthy primary HBEC donor (male, 54 years; PCS-300-010, Lot 70034736, ATCC, Manassas, VA, USA). HBEC and ePNEC cultures were plated and differentiated side-by-side on the same 24-well plates. Eighteen hours prior to stimulation, the basolateral medium of both the ePNEC and HBEC cultures was changed to an HBEC medium (#00193517, Lonza, Basel, Switzerland). Cultures were challenged with HDM (50 μL of 120 μg/mL corresponding to 6 μg total protein in PBS; LoTox *D*. *pteronyssinus* antigen, Indoor biotechnologies, Charlottesville, VA, USA) or control phosphate-buffered saline (PBS, Thermo Fisher Scientific, Waltham, MA, USA) at the apical surface at day 60 of culture. Conditioned basolateral media were collected from both cell types at 6h. In total, 20 samples were analyzed: HBEC (*n* = 5), HBEC + HDM *(n* = 5), ePNEC (*n* = 5), and ePNEC + HDM (*n* = 5). Each sample represented a single-well replicate sampled across three differentiation plates (two wells from plate 1, two wells from plate 2, and one well from plate 3).

### 2.2. Lactate Dehydrogenase (LDH) Assay

LDH activity in the basolateral supernatants was measured using the LDH-Glo Cytotoxicity Assay (#J2380, Promega, Madison, WI, USA). For each sample, 10 μL of basolateral media was collected and diluted 1:100 into an LDH storage buffer (200 mM Tris-HCl, pH 7.3, Sigma-Aldrich, St. Louis, MO, USA; 10% glycerol, Sigma-Aldrich, St. Louis, MO, USA; 1% BSA, Thermo Fisher Scientific, Waltham, MA, USA). Wells without cells were included as negative controls to determine the culture medium background. A maximum-release control was prepared by exposing matched cultures to 5% Triton-X100 (Thermo Fisher Scientific, Waltham, MA, USA) for 15 min. A spontaneous LDH control was taken from two unstimulated wells. For the assay, 50 μL of LDH Detection Reagent was mixed with 50 μL of diluted sample in an opaque-walled 96-well plate. Samples were recorded as technical duplicates and plates were incubated at room temperature for 30 min before luminescence was recorded (Synergy LX, BioTek, Winooski, VT, USA). Cytotoxicity was calculated using the following formula:

$$Cytotoxicity\;(\%)=\frac{Sample\;LDH-Spontaneous\;LDH}{Maximum\;LDH-Spontaneous\;LDH}\;\times 100$$

Assays were performed prior to submission of samples for metabolomics analysis (Table S1).

### 2.3. Metabolome Analysis Workflow

The workflow of metabolome analysis in this study included the following major steps: sample pre-treatment and normalization, chemical isotope labeling (CIL), LC-MS analysis, data processing and metabolite identification, and statistical analysis.

In the CIL LC-MS metabolome analysis, the whole metabolome was analyzed by targeting up to four submetabolomes or channels: amine/phenol-, carboxyl-, hydroxyl- and carbonyl-submetabolome [10]. The combined results from the four channels were able to provide a comprehensive profile of the entire metabolome, e.g., about 86% to 96% of the chemical space in various metabolome databases [10]. In each channel, after the sample pre-treatment step (e.g., protein precipitation, metabolite extraction, etc.), all samples were first derivatized with a pair of isotopic labeling reagents (i.e., ^12^C-/^13^C-reagents) prior to LC-MS analysis. The individual samples were labeled with ^12^C-reagents and a pooled sample, which was generated by mixing an aliquot from each individual sample, was labeled with ^13^C-reagents. After labeling, the ^12^C-labeled individual sample was mixed with the same amount of the ^13^C-labeled pool, followed by LC-MS analysis. In the mass spectra, each metabolite was detected as a peak pair, i.e., the light peak from the ^12^C-labeled individual sample and the heavy peak from the ^13^C-labeled pool. The peak intensity ratio between ^12^C-peak and ^13^C-peak represents the relative quantification result for a specific metabolite in an individual sample. Since the same ^13^C-labeled pooled sample was spiked into all the ^12^C-labeled individual samples, the ^12^C-/^13^C-peak ratio values for a specific metabolite across all individual samples reflected the metabolite’s concentration differences among them. The ^13^C-labeled pooled sample served as an internal reference to analyze all of the individual samples, correcting for matrix and ion suppression effects and instrument sensitivity drifts for accurate and precise quantification.

#### 2.3.1. Chemicals and Reagents

All chemicals and reagents were purchased from Sigma-Aldrich (Markham, ON, Canada), except those specifically stated. LC-MS-grade water, acetonitrile, methanol and formic acid were purchased from Canadian Life Sciences (Peterborough, ON, Canada). The metabolome quantification kit for sample normalization and the chemical isotope labeling kits for sample labeling were purchased from Nova Medical Testing Inc. (Edmonton, AB, Canada).

#### 2.3.2. Sample Pre-Treatment and Normalization

To minimize potential batch-related variability, samples were randomized prior to all experimental procedures to reduce intra-batch effects during sample analysis. Given the small number of samples, all samples were prepared and analyzed in a single batch and analytical sequence (i.e., there was no batch separation). Missing sample normalization was carried out by measuring the total metabolite concentration in each sample [11,12]. A proprietary metabolome quantification kit (Product Number: NMT-6001-KT) from Nova Medical Testing Inc. (Edmonton, AB, Canada) was used to measure the total concentration in an aliquot of 25 µL of the sample, following the standard operating procedure (SOP) provided in the kit. According to the quantification results, different volumes of the sample were taken and then dried down to adjust the samples to the same concentration. The samples were then stored in a −80 °C freezer and used for the following preparations and analyses.

#### 2.3.3. Chemical Isotope Labeling

Chemical isotope labeling of each sample was carried out by following the SOPs provided in the labeling kits (Nova Medical Testing Inc. (Edmonton, AB, Canada), Product Numbers: NMT-4101-KT, NMT-4123-KT, NMT-4145-KT, and NMT-4167-KT). For each labeling experiment, an aliquot of 25 µL of the sample was used. The amine-/phenol metabolites were labeled using a dansylation reaction [13]. The carboxyl metabolites were labeled using DMPA bromide [14]. The hydroxyl metabolites were labeled using a base-activated dansylation reaction [15]. The carbonyl metabolites were labeled using dansylhydrazine [16].

#### 2.3.4. Liquid Chromatography–Mass Spectrometry (LC-MS) Analysis

After labeling, the ^12^C_2_-labeled individual sample was mixed with the ^13^C_2_-labeled reference sample in equal volumes. The mixture was injected into LC-MS equipment for analysis. Before LC-MS analysis of the entire sample set, a quality control (QC) sample was prepared by an equal volume mix of a ^12^C-labeled and a ^13^C-labeled pooled sample. QC samples were run at an interval of one QC injection after 10 sample injections. All LC-MS analyses were carried out using a Thermo Vanquish LC linked to a Bruker Impact II QTOF Mass Spectrometer. The column used was an Agilent Eclipse Plus reversed-phase C18 column (150 × 2.1 mm, 1.8 µm particle size) and the column oven temperature was 40 °C. Mobile phase A was 0.1% (*v*/*v*) formic acid in water and mobile phase B was 0.1% (*v*/*v*) formic acid in acetonitrile. The gradient setting was t = 0 min, 25% B; t = 10 min, 99% B; t = 15 min, 99% B; t = 15.1 min, 25% B; t = 18 min, 25% B. The flow rate was 400 µL/min. Mass spectral acquisition rate was 1 Hz, with an m/z range from 220 to 1000.

#### 2.3.5. Data Processing and Metabolite Identification

All of the raw LC-MS data were first converted to .csv files using DataAnalysis (Bruker Daltonics, Bremen, Germany). The exported files were uploaded to IsoMS Pro (Nova Medical Testing Inc., Edmonton, AB, Canada.) for data processing and metabolite identification. The ^12^C-/^13^C-peak pairs in each sample were first extracted and the peak intensity ratio was calculated for each peak pair [17]. In this step, all of the redundant information was filtered out (e.g., adduct ions, dimers, etc.) to retain one peak pair for each metabolite. Then the same peak pair (i.e., metabolite) from different samples was aligned and the missing ratio values were filled back by the software. After that, data cleansing was carried out to remove peak pairs that originated from blank samples and that were not present in at least 80.0% of samples in any group. Data was then normalized by the Ratio of Total Useful Signal, calculated as the sum of all useful ^12^C-peaks over the sum of all useful ^13^C-peaks, served as post-acquisition normalization [11].

Metabolite identification was carried out using a three-tiered approach against NovaMT Metabolite Databases 2.0 (Nova Medical Testing Inc.) [10]. In tier 1, peak pairs were searched against a labeled metabolite library (CIL Library) based on accurate mass and retention time, comprising a positive identification. In tier 2, the remaining peak pairs were searched against a linked identity library (LI Library), which includes over 9000 pathway-related metabolites, providing high-confidence putative identification results based on accurate mass and predicted retention time matches. In tier 3, any remaining peak pairs were searched, based on accurate mass match, against the MyCompoundID (MCID) library (www.mycompoundid.org, accessed 1 August 2025) composed of 8021 known human endogenous metabolites (zero-reaction library), their predicted metabolic products from one metabolic reaction (375,809 compounds) (one-reaction library) and two metabolic reactions (10,583,901 compounds) (two-reaction library) [18]. For metabolite-level comparisons, equal variance was not assumed during statistical testing, and the data were not log-transformed before univariate analysis. To account for multiple hypothesis testing, false discovery rate (FDR) correction was applied across all metabolite-level comparisons, using a q-value threshold of 0.25. Both nominal *p*-values and FDR-adjusted q-values are reported in Supplementary Table S2.

### 2.4. Pathway Analysis

Pathway analysis was performed using a dedicated input file in which each metabolite was represented by a unique KEGG identifier. Isomeric features were consolidated at the pathway level through unique KEGG mapping, ensuring that no metabolite was counted more than once. Further, analysis was performed using tier 1 and tier 2 annotated metabolites only. The analysis was conducted in MetaboAnalyst 6.0 (https://www.metaboanalyst.ca/, accessed 10 January 2026) without data transformation, and auto-scaling was applied prior to analysis [19]. Pathway enrichment was carried out using the Global Test, and pathway topology was evaluated using relative betweenness centrality. All compounds in the selected homo sapiens (KEGG) pathway library were used as the reference metabolome. Pathway impact values were interpreted in the context of metabolite coverage and pathway mapping completeness.

### 2.5. Data Visualization and Statistics

Volcano plots were generated using the Volcano plot tool on usegalaxy.org (accessed 5 January 2026) [20,21]. Heatmaps were created using Morpheus (https://software.broadinstitute.org/morpheus, accessed 5 January 2026). Copilot (GenAI, Microsoft Corporation, Redmond, WA, USA, accessed 2 January 2026) was employed to generate the R code used to create the Venn diagrams and the bubble plot. All code was manually inspected, edited, and executed by the authors using ggplot2 in R Studio (2024.09.0, build 375, Posit Software, Boston, MA, USA). Overlap significance between metabolite lists was assessed using a hypergeometric test, with the background universe defined as all metabolites detected (*n* = 791). *p*-values were computed in R using the upper-tail hypergeometric distribution and are reported in the Results (Table 1).

## 3. Results

### 3.1. Global Metabolic Differences Between ePNEC and HBECs at Steady State and After HDM Stimulation

**Figure 1:**
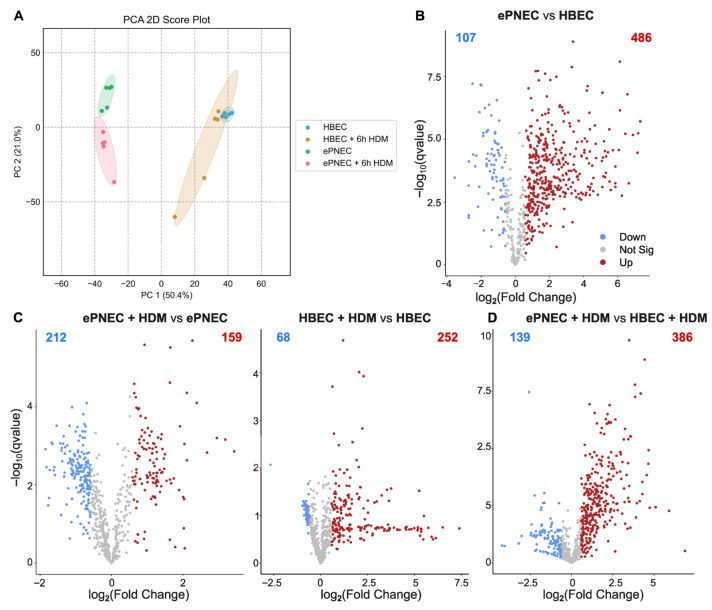
**Global metabolic landscape of ePNECs and HBECs.** (**A**) Principal component analysis (PCA) of all detected metabolites showing clear separation between Pulmonary neuroendocrine-enriched human airway epithelium (ePNECs) and Primary human bronchial epithelial cells (HBECs) along PC1 (50.4%), with House dust mites (HDM) stimulation driving additional within-cell-type variation along PC2 (21.0%). Each point represents an individual sample. (**B**–**D**) Volcano plots depicting log_2_FoldChange (FC) on the x-axis and –log_10_(qvalue) on the y-axis. Comparisons show ePNECs vs. HBECs at baseline (**B**), ePNEC + HDM vs. ePNEC, and HBEC + HDM vs. HBEC (**C**), and ePNEC + HDM vs. HBEC + HDM (**D**). Numbers and colored dots indicate significantly altered metabolites (FC > 1.5 and False Discovery Rate (FDR)-adjusted q-value threshold < 0.25).

Principal component analysis (PCA) revealed a strong separation between the ePNEC and HBEC cultures, indicating that culture type was the primary driver of metabolic variance (PC1 = 50.4%, Figure 1A). HDM stimulation induced additional within-cell-type shifts along PC2 (21.0%), with both ePNECs and HBECs showing consistent movement away from their respective PBS controls. Of note, the heterogeneity of the samples was higher in the stimulated HBEC cells. LDH measurements confirmed that the stimulation protocol did not induce cytotoxicity, with LDH release remaining below 0.1% in both HDM- and PBS-treated cultures (Table S1). These patterns demonstrate that ePNECs and HBECs possess distinct baseline metabolic profiles and mount cell culture-specific responses to HDM. Subsequent Volcano analysis showed that a substantial number of metabolites show expression changes between conditions (FC > 1.5 and FDR-adjusted q-value threshold < 0.25). At steady state, 486 and 107 metabolites were more and less expressed in ePNEC compared to HBEC, respectively (Figure 1B, Table S2). Comparing HDM-stimulated cells with PBS controls, the number of metabolites up-regulated was higher in the HBECs (252) than in the ePNECs (159), while the number of down-regulated metabolites showed the opposite trend: 212 and 68 metabolites were down-regulated in ePNEC and HBEC after HDM stimulation, respectively (Figure 1C). Lastly, a differential expression analysis between the stimulated ePNEC and HBEC showed 386 metabolites with increased levels in ePNEC (Figure 1D). Overall, the magnitude and direction of fold changes highlighted that ePNECs displayed broader and more pronounced metabolite expression changes than HBECs.

### 3.2. Overlap of Differentially Abundant Metabolites

**Figure 2:**
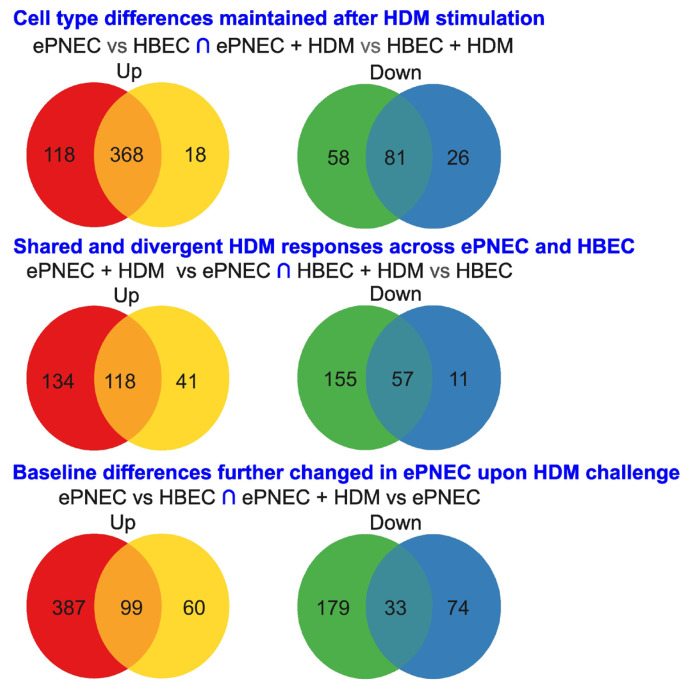
**Venn diagrams showing the overlap of differentially abundant metabolites.** Numbers indicate the count of up-regulated (**left**) and down-regulated (**right**) metabolites in each region. Venn diagrams are color-coded by direction of regulation. Up-regulated gene sets are shown in red and yellow, with shared genes indicated in orange. Down-regulated gene sets are shown in blue and green, with overlapping genes shown in teal. Overlap significance was assessed using a hypergeometric test (Table 1) with a background universe of 791 detected metabolites.

We next analyzed the overlap of differentially abundant metabolite sets reflecting cell type differences maintained after HDM stimulation (ePNEC vs. HBEC **∩** ePNEC + HDM vs. HBEC + HDM), shared and divergent HDM responses across ePNEC and HBEC (ePNEC + HDM vs. ePNEC **∩** HBEC + HDM vs. HBEC), and baseline differences further changed in ePNEC upon HDM challenge (ePNEC vs. HBEC **∩** ePNEC + HDM vs. ePNEC) (Figure 2, Table S3). Venn analyses showed strong preservation of baseline metabolic differences after HDM exposure, with substantial overlaps in both up-regulated (368 shared) and down-regulated metabolites (81 shared). HDM stimulation also elicited a considerable shared response across cell types, including 118 shared up-regulated and 57 shared down-regulated metabolites. Finally, baseline ePNEC–HBEC differences partially overlapped with the ePNEC-specific HDM response, with 99 shared up-regulated and 33 shared down-regulated metabolites. To determine whether the observed overlaps reflected coordinated biological regulation rather than random coincidence, we quantified overlap significance using a hypergeometric test (Table 1, Table S3). The overlap between baseline cell culture differences (ePNEC vs. HBEC) and HDM-stimulated differences (ePNEC + HDM vs. HBEC + HDM) was extremely significant for both up-regulated (*p* = 7.0 × 10^−93^) and down-regulated metabolites (*p* = 2.8 × 10^−49^), indicating that core metabolic distinctions between ePNECs and HBECs are strongly preserved following allergen exposure. HDM responses also showed substantial shared regulation across cell types, with significant overlaps between ePNEC + HDM vs. ePNEC and HBEC +HDM vs. HBEC for both up-regulated (*p* = 1.8 × 10^−35^) and down-regulated metabolites (*p* = 5.8 × 10^−25^). These results support the presence of common metabolites activated by HDM exposure in both cell cultures, which is independent of the presence of PNECs. Finally, the overlap between baseline ePNEC-HBEC differences and the ePNEC-specific HDM response was significant for up-regulated metabolites (*p* = 7.8 × 10^−9^), but not for down-regulated features (*p* = 0.18). This pattern suggests that pre-existing metabolic distinctions in ePNECs preferentially predispose toward enhanced up-regulation upon allergen challenge, rather than coordinated down-regulation. These metabolites might hence be important for cell identity preservation.

### 3.3. Detected Metabolite Classes and Specific Metabolite Level Changes

**Figure 3:**
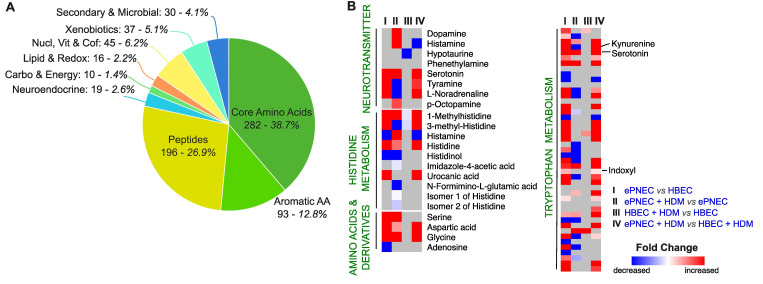
**Metabolic class—level differences between ePNECs and HBECs.** (**A**) Pie chart showing the distribution of Kyoto Encyclopedia of Genes and Genomes (KEGG) annotated metabolite classes with number of metabolites and percentage of total annotated metabolite classes. (**B**) Heatmap of selected metabolites across four comparisons: (I) ePNEC vs. HBEC, (II) ePNEC + HDM vs. ePNEC, (III) HBEC + HDM vs. HBEC, and (IV) ePNEC + HDM vs. HBEC + HDM. Metabolites are grouped by pathway: neurotransmitters, histidine metabolism, amino acids and derivatives, and tryptophan metabolism. Color scale indicates significant log_2_ fold change (red = increased, blue = decreased, gray = no significant fold change with criteria FC > 1.2 and False Discovery Rate (FDR)-adjusted q-value threshold < 0.25). AA = amino acids; carbo = carbohydrate; cof = cofactor; ePNEC = epithelial-derived pulmonary neuroendocrine cells; HBEC = human bronchial epithelial cells; HDM = House dust mites; nucl = nucleotides; vit = vitamins; vs. = versus.

Metabolite annotation from KEGG allowed us to create nine functional clusters for all of the detected compounds (Table S4). The clusters showed that the detected features spanned multiple biochemical classes, with core amino acids (282, 38.7%) and peptides (196, 26.9%) representing the largest fractions, followed by aromatic amino acids (93, 12.8%), nucleotides, vitamins and cofactors, xenobiotics and their environments, secondary and microbial metabolites, neuroendocrine-related compounds, and a smaller contribution from carbohydrate and energy and lipid and redox pathways (Figure 3A). This distribution indicates that the dataset is strongly enriched for amino acid-centered metabolism and peptide derivatives. Of note, many canonical neurotransmitters, including dopamine, serotonin, histamine, GABA, and catecholamines, are derived directly from amino acids such as tyrosine, tryptophan, histidine, and glutamate. Aromatic amino acids (12.8%), which include the precursors for monoamine neurotransmitters, represented another substantial fraction.

To examine how specific metabolites change in expression between our conditions, we created heatmaps highlighting significant changes (FC > 1.2 and FDR-adjusted q-value threshold < 0.25) in color for neurotransmitters, amino acids and derivatives, and compounds important for the histidine and tryptophan metabolism, respectively (Figure 3B, gray color indicates no significant change). ePNEC cultures consistently exhibited higher levels of several secreted neurotransmitters at baseline, highlighting their intrinsic neuroendocrine signature: serotonin (FC = 45.6, q = 3.7 × 10^−6^), tyramine (FC = 3.3, q = 4.9 × 10^−5^), and L-noradrenaline (FC = 3.4, q = 2.1 × 10^−7^, tier 2). Other neurotransmitters were only elevated in ePNEC after HDM challenge, namely dopamine (FC = 3.7, q = 6.6 × 10^−3^), histamine (FC = 3.3, q = 4.7 × 10^−3^), and p-octopamine (FC = 1.8, q = 2.4 × 10^−3^, tier 2). Interestingly, some compounds of the histidine pathway were elevated at baseline in the basolateral media in ePNEC compared to HBEC, such as histidine (FC = 58.5, q = 1.5 × 10^−4^) and urocanic acid (FC = 6.1, q = 2.7 × 10^−5^). Others are significantly more abundant in the media only after HDM stimulation, suggesting that the latter compounds are involved in the cellular response after allergen exposure, e.g., histamine (FC = 3.3, q = 4.7 × 10^−3^) and imidazole-4-ascetic acid (FC = 1.4, q = 5.9 × 10^−3^). Several amino acids are significantly elevated in the basolateral media in ePNEC compared to HBEC, e.g., serine (FC = 12.6, q = 7.0 × 10^−5^), aspartic acid (protonated aspartate, FC = 14.8, q = 1.7 × 10^−5^), glycine (FC = 8.5, q = 1.4 × 10^−6^), and tryptophan (FC = 52.8, q = 1.2 × 10^−5^), the precursor for both serotonin and kynurenine, two pathways with major neuroimmune relevance. ePNECs further showed higher secreted levels of serotonin, kynurenine (FC = 25.3, q = 3.1 × 10^−6^) and indoxyl (FC = 1.5, q = 4.0 × 10^−6^, tier 2), indicating enhanced tryptophan catabolism through both the serotonin and kynurenine branches.

### 3.4. MetaboAnalyst Pathway Analysis

**Figure 4:**
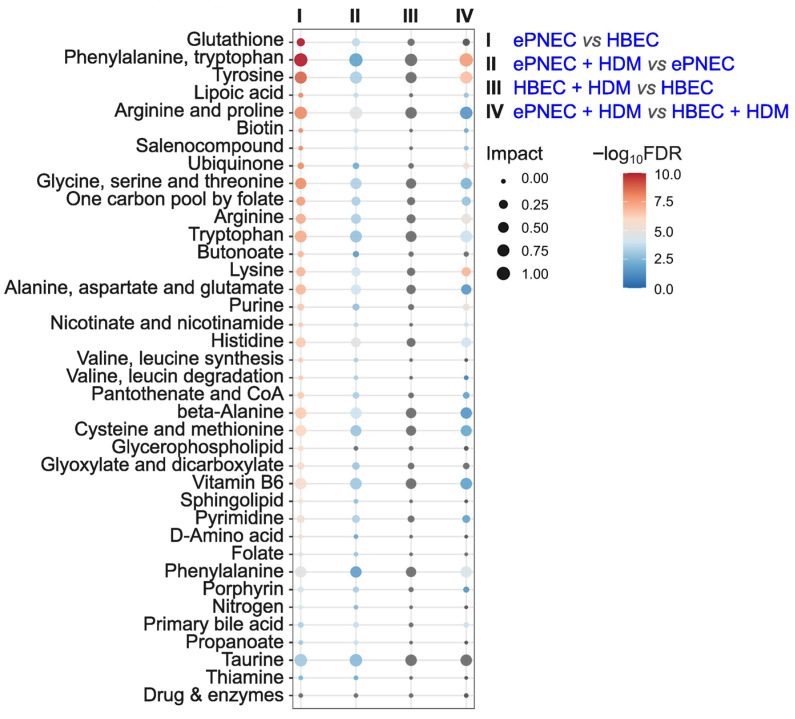
**MetaboAnalyst pathway analysis of differentially abundant metabolites.** Dot plots show statistically enriched metabolism pathways across four comparisons: (I) ePNEC vs. HBEC, (II) ePNEC + HDM vs. ePNEC, (III) HBEC + HDM vs. HBEC, and (IV) ePNEC + HDM vs. HBEC + HDM. Dot size reflects pathway impact, and color indicates statistical significance of the pathway (−log_10_FDR, FDR < 0.05) with gray color representing non-significance. All pathways shown are classified as metabolism pathways in MetaboAnalyst. ePNEC = epithelial-derived pulmonary neuroendocrine cells; HBEC = Human bronchial epithelial cells; HDM = House dust mites; FDR = False Discovery Rate.

To dive deeper into biological pathway differences, we used MetaboAnalyst for differentially abundant metabolites (Figure 4, Table S5). The bubble plot in Figure 4 displays enriched pathways across four comparisons: (I) ePNEC vs. HBEC, (II) ePNEC + HDM vs. ePNEC, (III) HBEC + HDM vs. HBEC, and (IV) ePNEC + HDM vs. HBEC + HDM. Strikingly, most of the pathways were significant (FDR < 0.05) in conditions I, II and IV to varying degrees, while none of the pathways were significant in the HBEC comparison of HDM-stimulated vs. naïve cells. This indicates that, although HBECs exhibit a substantial number of differentially abundant metabolites after HDM exposure, these changes are more diffusely distributed across pathways and do not converge into significant, high-impact pathway-level enrichment. In contrast, ePNECs show both extensive metabolite-level changes and significant pathway alterations (comparison I) including in the phenylalanine, tyrosine, and tryptophan pathways, which are directly linked to neurotransmitter biosynthesis [22]. Another significant hit with reasonable impact is the glutathione pathway, which is essential for maintaining the redox environment and cofactor availability required for monoamine synthesis [23]. Also, the arginine and proline pathways are significantly different in ePNEC compared to HBEC. Arginine is known to fuel nitric oxide and polyamine synthesis, while proline interconverts with glutamate and contributes to redox regulation, providing metabolic support for neurotransmitter production and epithelial stress responses [24,25]. This pathway was the most significantly altered one between ePNEC + HDM vs. ePNEC (*p* = 2.52 × 10^−5^), suggesting that allergen exposure further amplifies these neuroendocrine-linked mechanisms. In comparison, fewer pathways were affected between ePNEC + HDM vs. HBEC + HDM, with phenylalanine, tryptophan, tyrosine and lysine metabolism pathways being the predominantly significant and impactful ones (IV, Figure 4). These findings collectively show that ePNECs engage a broader and more neuroactive metabolic program than HBECs, both at baseline and in response to allergen challenge, highlighting that further investigation on the role of PNECs in response to inhaled stimuli such as allergens is warranted.

## 4. Discussion

This study provides a comprehensive metabolic comparison of ePNECs and HBECs at steady state and following stimulation with the common aeroallergen HDM, revealing fundamental differences between the cultures and stimulus-responsive pathways. Across all analyses, culture type emerged as the dominant determinant of metabolic variance, with PCA showing a clear separation between ePNECs and HBECs and the differential abundance analyses highlighting widespread baseline differences. These findings reinforce the value of the novel ePNEC in vitro model for studying neuroendocrine-linked biology of the airway epithelium in response to environmental challenges. Compared with HBECs, ePNECs displayed broader and more pronounced changes in metabolite abundance, both at baseline and after HDM exposure. This heightened responsiveness aligns with the emerging view that PNECs act as sentinels of the inhaled environment resulting in potent metabolic and signaling outputs upon allergen challenge. The strong preservation of baseline metabolic differences after HDM stimulation suggests that allergen exposure does not override intrinsic cell-type identity but instead builds upon pre-existing metabolic signatures.

Overlap analyses provided additional insight into how these intrinsic features shape allergen-induced responses. The extremely significant overlap between baseline and HDM-stimulated comparisons indicates that core metabolite expression differences between ePNECs and HBECs remain stable under allergen challenge. At the same time, both cell types shared substantial HDM-induced metabolite-level shifts, reflecting a potentially conserved PNEC-independent response to HDMs. This response may be mediated by the co-cultured cell types such as the goblet cells, club cells, basal cells, ciliated epithelial cells, and myofibroblasts known to be present in the cultures [8].

Functional annotation revealed strong enrichment of detected metabolites for amino acids, peptides, and aromatic amino acids. These are biochemical classes that form the backbone of neurotransmitter biosynthesis. Heatmap analyses showed that some neurotransmitters were elevated at baseline in the ePNECs (serotonin, tyramine, L-noradrenaline), while others showed increased abundance in basolateral media only after HDM stimulation (dopamine, histamine, p-octopamine), indicating that allergen exposure selectively engages specific neurotransmitters.

A systematic pathway analysis with MetaboAnalyst in addition to functional annotation of each detected metabolite reinforced the conclusion that ePNECs engage a more coordinated and neuroactive metabolic program than HBECs. At baseline, ePNECs showed strong enrichment in amino acid pathways (phenylalanine, tyrosine, and tryptophan metabolism) that directly support monoamine neurotransmitter biosynthesis. Additionally, pathways that do not contain classical neurotransmitter precursors but provide essential metabolic support for neuroendocrine signaling (glutathione, arginine and proline) were also altered. The maintained enrichment of the arginine and proline pathway in ePNECs after HDM exposure suggests that allergen stimulation amplifies these neuroendocrine-linked mechanisms. In contrast, HBECs had numerous differentially abundant metabolites, but did not exhibit significant pathway-level enrichment, indicating that their HDM-induced changes are more diffusely distributed and possibly lack the coordinated pathway remodeling observed in ePNECs. The persistent differences between HDM-stimulated ePNECs and HBECs highlight that allergen exposure reinforces, rather than blurs, the metabolic divergence between these cell culture types.

Beyond characterizing these differences, the dataset provides a valuable resource for hypothesis generation. Prior studies of airway neuroendocrine biology have typically focused on a small number of 2–3 single neurotransmitters [6,26]. In contrast, the metabolomics approach used here enables a comprehensive screening of all detectable metabolites that directly represent a neurotransmitter or the corresponding precursor. Yet some limitations should be acknowledged. Untargeted metabolomics does not capture all biochemical classes with equal sensitivity. Larger or low-abundance metabolites may be under-represented or undetected. Some pathways such as lipid mediators and complex carbohydrates may be less comprehensively covered by our approach. Additionally, metabolite identification relies on database matching and may miss rare or novel compounds. These limitations do not diminish the value of the dataset but highlight the need for complementary targeted assays to validate and extend key findings.

In summary, this study demonstrates that a human airway epithelium enriched with PNECs possesses a distinct metabolic identity, characterized by an increase in neuroactive compounds, which are altered in response to the aeroallergen HDM. The breadth of the metabolic differences uncovered here underscores the utility of the novel in vitro ePNEC model for studying the neuroendocrine response of the airways to inhaled allergens and provides a foundation for future mechanistic work.

## 5. Conclusions

The PNEC-enriched human airway epithelium (ePNEC) exhibits a distinct and highly coordinated neuroactive metabolic program that persists and intensifies following HDM exposure, underscoring the unique sensory and signaling capacity of these rare epithelial cells. By integrating global metabolomic profiling with pathway-level analyses, this study reveals that ePNECs not only maintain a stable neuroendocrine-linked metabolic identity but also mount amplified, neurotransmitter-centered responses to allergen challenge. These findings establish ePNECs as a powerful in vitro model for dissecting neuroimmune–epithelial interactions and provide a metabolic framework that can guide future mechanistic studies aimed at understanding how PNECs shape airway physiology and allergic inflammation.

## Figures and Tables

**Table 1:** Hypergeometric test for overlap significance between differentially abundant metabolite sets.

Comparison Pair	Regulation	Hypergeometric *p*-Value
ePNEC vs. HBEC **∩** ePNEC + HDM vs. HBEC + HDM	Up	7.00 × 10^−93^
	Down	2.85 × 10^−49^
ePNEC + HDM vs. ePNEC **∩** HBEC + HDM vs. HBEC	Up	1.76 × 10^−35^
	Down	5.80 × 10^−25^
ePNEC vs. HBEC **∩** ePNEC + HDM vs. ePNEC	Up	7.81 × 10^−9^
	Down	0.1841

## Data Availability

The original data presented in the study are openly available in MetaboLights (https://www.ebi.ac.uk/metabolights/MTBLS13750, accessed on 23 January 2026).

## Supplementary Materials

The following supporting information can be downloaded at https://www.mdpi.com/article/10.3390/metabo16020137/s1, Table S1: LDH assay; Table S2: All Metabolite changes; Table S3: Venn diagram metabolites and Hypergeometric test values; Table S4: Metabolite super-classification for Figure 3A; Table S5: MetaboAnalyst Pathway Analyses.

## Abbreviations

The following abbreviations are used in this manuscript:

ALI	Air–liquid interface
CGRP	Calcitonin gene-related peptide
CIL	Chemical isotope labeling library
ePNEC	Pulmonary neuroendocrine-enriched human airway epithelium
FC	Fold Change
FDR	False Discovery Rate
GABA	γ-Aminobutyric acid
GM-CSF	Granulocyte–Macrophage colony-stimulating factor
HBECs	Primary human bronchial epithelial cells
HDM	House dust mite
ILC2s	Group 2 innate lymphoid cells
KEGG	Kyoto Encyclopedia of Genes and Genomes
LDH	Lactate dehydrogenase
LC–MS	Liquid chromatography–mass spectrometry
LI	Linked-identity library
MCID	MyCompoundID
PAR1	Protease-activated receptor 1
PBS	Phosphate-buffered saline
PCA	Principal component analysis
PNECs	Pulmonary neuroendocrine cells
QC	Quality control
QTOF	Quadrupole time-of-flight
TMIC	The Metabolomics Innovation Centre
UHPLC	Ultra-high-performance liquid chromatography

## References

[1] Scheuermann D.W. (1997). Comparative histology of pulmonary neuroendocrine cell system in mammalian lungs. Microsc. Res. Tech..

[2] Yabumoto Y., Watanabe M., Ito Y., Maemura K., Otsuki Y., Nakamura Y., Yanagawa Y., Obata K., Watanabe K. (2008). Expression of GABAergic system in pulmonary neuroendocrine cells and airway epithelial cells in GAD67-GFP knock-in mice. Med. Mol. Morphol..

[3] Dey R.D., Hoffpauir J.M. (1986). Ultrastructural colocalization of the bioactive mediators 5-hydroxytryptamine and bombesin in endocrine cells of human fetal airways. Cell Tissue Res..

[4] Cutz E. (1982). Neuroendocrine cells of the lung. An overview of morphologic characteristics and development. Exp. Lung Res..

[5] Dey R.D., Snyder J.M., Speciale S.G., Price J. (1986). Characterization of human pulmonary endocrine cells maintained in vitro. Exp. Lung Res..

[6] Sui P., Wiesner D.L., Xu J., Zhang Y., Lee J., Van Dyken S., Lashua A., Yu C., Klein B.S., Locksley R.M. (2018). Pulmonary neuroendocrine cells amplify allergic asthma responses. Science.

[7] Deprez M., Zaragosi L.E., Truchi M., Becavin C., Ruiz Garcia S., Arguel M.J., Plaisant M., Magnone V., Lebrigand K., Abelanet S. (2020). A Single-Cell Atlas of the Human Healthy Airways. Am. J. Respir. Crit. Care Med..

[8] Mann-Nuttel R., Mandal S., Armbruster M., Puttagunta L., Forsythe P. (2025). Human Pulmonary Neuroendocrine Cells Respond to House Dust Mite Extract With PAR-1 Dependent Release of CGRP. Allergy.

[9] Seo S.K., Kwon B. (2023). Immune regulation through tryptophan metabolism. Exp. Mol. Med..

[10] Zhao S., Li H., Han W., Chan W., Li L. (2019). Metabolomic Coverage of Chemical-Group-Submetabolome Analysis: Group Classification and Four-Channel Chemical Isotope Labeling LC-MS. Anal. Chem..

[11] Wu Y., Li L. (2016). Sample normalization methods in quantitative metabolomics. J. Chromatogr. A.

[12] Wu Y., Li L. (2012). Determination of total concentration of chemically labeled metabolites as a means of metabolome sample normalization and sample loading optimization in mass spectrometry-based metabolomics. Anal. Chem..

[13] Guo K., Li L. (2009). Differential 12C-/13C-isotope dansylation labeling and fast liquid chromatography/mass spectrometry for absolute and relative quantification of the metabolome. Anal. Chem..

[14] Guo K., Li L. (2010). High-performance isotope labeling for profiling carboxylic acid-containing metabolites in biofluids by mass spectrometry. Anal. Chem..

[15] Zhao S., Luo X., Li L. (2016). Chemical Isotope Labeling LC-MS for High Coverage and Quantitative Profiling of the Hydroxyl Submetabolome in Metabolomics. Anal. Chem..

[16] Zhao S., Dawe M., Guo K., Li L. (2017). Development of High-Performance Chemical Isotope Labeling LC-MS for Profiling the Carbonyl Submetabolome. Anal. Chem..

[17] Zhou R., Tseng C.L., Huan T., Li L. (2014). IsoMS: Automated processing of LC-MS data generated by a chemical isotope labeling metabolomics platform. Anal. Chem..

[18] Li L., Li R., Zhou J., Zuniga A., Stanislaus A.E., Wu Y., Huan T., Zheng J., Shi Y., Wishart D.S. (2013). MyCompoundID: Using an evidence-based metabolome library for metabolite identification. Anal. Chem..

[19] Pang Z., Xu L., Viau C., Lu Y., Salavati R., Basu N., Xia J. (2024). MetaboAnalystR 4.0: A unified LC-MS workflow for global metabolomics. Nat. Commun..

[20] Afgan E., Baker D., van den Beek M., Blankenberg D., Bouvier D., Cech M., Chilton J., Clements D., Coraor N., Eberhard C. (2016). The Galaxy platform for accessible, reproducible and collaborative biomedical analyses: 2016 update. Nucleic Acids Res..

[21] Wickham H. (2016). ggplot2: Elegant Graphics for Data Analysis; Use R!.

[22] Fernstrom J.D., Fernstrom M.H. (2007). Tyrosine, phenylalanine, and catecholamine synthesis and function in the brain. J. Nutr..

[23] Schafer F.Q., Buettner G.R. (2001). Redox environment of the cell as viewed through the redox state of the glutathione disulfide/glutathione couple. Free Radic. Biol. Med..

[24] Palmer R.M., Ashton D.S., Moncada S. (1988). Vascular endothelial cells synthesize nitric oxide from L-arginine. Nature.

[25] Krishnan N., Dickman M.B., Becker D.F. (2008). Proline modulates the intracellular redox environment and protects mammalian cells against oxidative stress. Free Radic. Biol. Med..

[26] Gu X., Karp P.H., Brody S.L., Pierce R.A., Welsh M.J., Holtzman M.J., Ben-Shahar Y. (2014). Chemosensory functions for pulmonary neuroendocrine cells. Am. J. Respir. Cell Mol. Biol..

